# Snoring Sounds Predict Obstruction Sites and Surgical Response in Patients with Obstructive Sleep Apnea Hypopnea Syndrome

**DOI:** 10.1038/srep30629

**Published:** 2016-07-29

**Authors:** Li-Ang Lee, Yu-Lun Lo, Jen-Fang Yu, Gui-She Lee, Yung-Lun Ni, Ning-Hung Chen, Tuan-Jen Fang, Chung-Guei Huang, Wen-Nuan Cheng, Hsueh-Yu Li

**Affiliations:** 1 Department of Otorhinolaryngology - Head and Neck Surgery, Sleep Center, Linkou-Chang Gung Memorial Hospital, Tao-Yuan 33305, Taiwan, ROC; 2 Faculty of Medicine, College of Medicine, Chang Gung University, Taoyuan 33303, Taiwan, ROC; 3 Department of Thoracic Medicine, Sleep Center, Linkou-Chang Gung Memorial Hospital, Tao-Yuan 33305, Taiwan, ROC; 4 Graduate Institute of Medical Mechatronics, Taiouan Interdisciplinary Otolaryngology Laboratory, Chang Gung University, Taoyuan 33303, Taiwan; 5 Faculty of Medicine, School of Medicine, National Yang-Ming University, Taipei 11221, Taiwan, ROC; 6 Department of Otolaryngology, Taipei City Hospital, Ren-Ai Branch, Taipei 10629, Taiwan, ROC; 7 Department of Chest Medicine, Taichung Tzu Chi Hospital, Buddhist Tzu Chi Medical Foundation, Taichung 42743, Taiwan, ROC; 8 Department of Laboratory Medicine, Linkou-Chang Gung Memorial Hospital, Tao-Yuan 33305, Taiwan, ROC; 9 Department of Medical Biotechnology and Laboratory Science, College of Medicine, Chang Gung University, Tao-Yuan 33303, Taiwan, ROC; 10 Graduate Institute of Biomedical Sciences, College of Medicine, Chang Gung University, Tao-Yuan 33303, Taiwan, ROC; 11 Department of Sports Sciences, University of Taipei, Tai-Pei 11153, Taiwan, ROC; 12 Department of Sleep Medicine, Royal Infirmary of Edinburgh, Edinburgh EH16 4SA, UK

## Abstract

Snoring sounds generated by different vibrators of the upper airway may be useful indicators of obstruction sites in patients with obstructive sleep apnea hypopnea syndrome (OSAHS). This study aimed to investigate associations between snoring sounds, obstruction sites, and surgical responses (≥50% reduction in the apnea-hypopnea index [AHI] and <10 events/hour) in patients with OSAHS. This prospective cohort study recruited 36 OSAHS patients for 6-hour snoring sound recordings during in-lab full-night polysomnography, drug-induced sleep endoscopy (DISE), and relocation pharyngoplasty. All patients received follow-up polysomnography after 6 months. Fifteen (42%) patients with at least two complete obstruction sites defined by DISE were significantly, positively associated with maximal snoring sound intensity (40–300 Hz; odds ratio [OR], 1.25, 95% confidence interval [CI] 1.05–1.49) and body mass index (OR, 1.48, 95% CI 1.02–2.15) after logistic regression analysis. Tonsil obstruction was significantly, inversely correlated with mean snoring sound intensity (301–850 Hz; OR, 0.84, 95% CI 0.74–0.96). Moreover, baseline tonsil obstruction detected by either DISE or mean snoring sound intensity (301–850 Hz), and AHI could significantly predict the surgical response. Our findings suggest that snoring sound detection may be helpful in determining obstruction sites and predict surgical responses.

Snoring is one of the most common symptoms of obstructive sleep apnea hypopnea syndrome (OSAHS)^1^. It is an annoying sound produced by vibrations of the soft tissues in the upper respiratory tract as a result of partial narrowing of these soft tissues during sleep^2^. Loud snoring is regarded to be noise pollution and often drives the patient to seek medical help^3^. A patient’s perception of his/her snoring may be inaccurate, and it should be measured objectively^4^. Although polysomnography (PSG) is the current gold standard for the diagnosis of OSAHS, it provides little information on snoring and is therefore used less in the clinical treatment of snoring. Accordingly, new technologies have been developed for the accurate detection of complex snoring events^5^,^6^, specific discriminations between primary snoring and OSAHS^7^,^8^, and precise estimation of OSAHS severity^9^,^10^.

Identification of the site of upper airway obstruction in OSAHS may be beneficial when deciding on other treatment than continuous positive airway pressure (CPAP) therapy. Moreover, the failure to identify and treat all levels of airway obstruction is a key reason for disappointing surgical results^11^. For example, patients with hypopharyngeal obstructions have worse outcomes of uvulopalatopharyngoplasty^12^ but better results of hypopharyngeal surgery^13^. Therefore, several clinical tools have been developed to assess upper airway obstructions such as the Friedman stage system (oropharyngeal anatomic classification)^14^, nasopharyngoscopy with the Müller manoeuvre^15^, upper airway pressure measurement^16^, magnetic resonance imaging (MRI)^17^, and drug-induced sleep endoscopy (DISE)^18^.

Previous studies on obstruction sites and acoustic analysis of snoring sounds have demonstrated that an obstruction level above the free margin of the soft palate produces a characteristic frequency and energy in the low frequency domain (Fig. 1), whereas an obstruction level below the free margin of the soft palate generates a characteristic frequency and energy in the high frequency domain (Fig. 2)^19^. Therefore, we hypothesized that complex snoring sounds are related to multi-level obstruction. The aims of this prospective study were to (1) examine associations between acoustic parameters of whole night snoring sounds during natural sleep and obstruction sites (multi-level and other levels) defined by DISE, and (2) verify the effects of these variables on surgical responses in patients with OSAHS.

## Results

### Study population

Thirty-four men and two women with a median age of 39 years were included in this study. More than half of them were overweight, had a thick neck, normal-sized tonsils, normal tongue position, Friedman’s anatomic stage 2, severe snoring, excessive daytime sleepiness, severe OSAHS, and decreased mean/minimal arterial oxygen saturation (SaO_2_; Table 1). Table 2 demonstrates the distribution of acoustic parameters of 6-hour snoring sounds during natural sleep. The obstruction sites were (in descending order of prevalence) the velum, oropharynx, tongue base, and epiglottis. Fifteen (42%) participants had multi-level obstructions and 21 (58%) had simple velopharynx obstruction (Table 3).

### Comparisons between multi-level obstruction and simple velopharynx obstruction

Compared to the participants with simple velopharynx obstruction, those with multi-level obstructions had significantly higher body mass index (BMI) and apnea-hypopnea index (AHI), and lower mean SaO_2_ and minimal SaO_2_ (Table 1). Moreover, total-, B1-, and B3- peak sound frequency (Fpeak), B2- and B3-snoring index (SI), total-, B1-, and B2-maximal sound intensity (Imax), and total-, B1-, and B2-mean sound intensity (Imean) of the those with multi-level obstructions were significantly different compared to those with simple velopharynx obstruction (Table 2).

### Correlations of patient characteristics with DISE findings

BMI, AHI, mean SaO_2_, and minimal SaO_2_ were significantly associated with multi-level obstructions (Table 4). AHI, mean SaO_2_, and minimal SaO_2_ were significantly correlated with velopharynx obstruction. Age, AHI, mean SaO_2_, and minimal SaO_2_ were significantly associated with lateral oropharyngeal wall obstructions, and AHI, mean SaO_2_, and minimal SaO_2_ were significantly associated with epiglottitis obstructions. None of the patient characteristics were significantly associated with tonsil or and tongue base obstructions.

### Correlations of snoring sound parameters with DISE findings

Table 5 demonstrates the associations of snoring sound parameters with obstruction sites. Total-, B1-, and B3-Fpeak, B2- and B3-SI, total-, B1-, and B2-Imax, and total-, B1-, and B2-Imean were significantly associated with multi-level obstructions. B1- and B2-Fpeak, and B3-SI were significantly correlated with partial-to-complete velopharynx obstructions, and B2-Imean and B3-mean sound frequency (Fmean) were significantly associated with partial-to-complete tonsil obstructions. Total-Fpeak, B1-, and B2-Imax, total-, B2-, and B3-Imean, and B3-Fmean were significantly associated with partial-to-complete lateral oropharyngeal wall obstructions. B3-Fpeak and B3-SI were significantly correlated with complete tongue base obstructions, and B1-Fpeak, B2-SI, B1-Imax, and B2-Imax were significantly associated with partial-to-complete epiglottitis obstructions.

### Patient characteristics, snoring sound parameters, and obstruction sites

#### Multi-level obstructions

In multivariate analysis, BMI (odds ratio [OR], 1.48, 95% confidence interval [CI] 1.02–2.15) and B1-Imax (OR, 1.25, 95% CI 1.05–1.49) were independent predictors of multi-level obstructions (Table 6). We determined the optimal cut-off values of these variables using receiver operating characteristic (ROC) curve. Therefore, participants with a higher BMI (≥25.6 kg/m^2^) were 7.21 (95% confidence intervals [CI] 1.14 to 45.6) times more likely to have multi-level obstructions relative to participants with a lower BMI (<25.6 kg/m^2^) after adjusting for B1-Imax (*p* = 0.036). Participants with a higher B1-Imax (≥60 dB) were 31.84 (95% CI 3.01 to 337.2) times more likely to have multi-level obstructions relative to participants with a lower B1-Imax (<60 dB) after adjusting for BMI (*p* = 0.004). B1-Imax was more sensitive and specific than BMI for predicting multi-level obstructions.

#### Velopharynx obstruction

B2-SI (OR, 1.01, 95% CI 1.00–1.02) was an independent predictor of complete velopharynx obstructions. Participants with a higher B2-SI (≥28 events/h) were 7.86 (95% CI 1.65 to 37.4) times more likely to have complete velopharynx obstruction relative to participants with a lower B2-SI (<28 events/h; *p* = 0.010).

#### Tonsil obstruction

B2-Imean (OR, 0.84, 95% CI 0.74–0.96) was an independent predictor of partial-to-complete tonsil obstructions. Participants with a higher B2-Imean (≥48 dB) were 0.18 (95% CI 0.04 to 0.78) times more likely to have tonsil obstructions relative to participants with a lower B2-Imean (<48 dB; *p* = 0.022).

#### Lateral oropharyngeal wall obstruction

B2-Imean (OR, 1.22, 95% CI 1.03–1.46) and B3-Fmean (OR, 1.01, 95% CI 1.00–1.02) were independent predictors of partial-to-complete lateral oropharyngeal wall obstructions. Participants with a higher B2-Imean (≥48 dB) were 40.28 (95% CI 2.75 to 590.8) times more likely to have lateral oropharyngeal wall obstructions relative to participants with a lower B2-Imean (<48 dB) after adjusting for B3-Fmean (*p* = 0.007). Participants with a higher B3-Fmean (≥1220 Hz) were 1.29 (95% CI 1.06 to 1.59) times more likely to have lateral oropharyngeal wall obstructions relative to participants with a lower B3-Fmean (<1220 Hz) after adjusting for B2-Imean (*p* = 0.013).

*Tongue base obstruction*. None of the patient characteristics or snoring sound parameters were independent predictors of partial-to-complete tongue base obstructions (data not shown). Accordingly, we investigated associations between complete tongue base obstructions and other variables, and found that B3-Fpeak (OR, 1.00, 95% CI 1.00–1.01) was an independent predictor of complete tongue base obstructions. Participants with a higher B3-Fpeak (≥1775 Hz) were 4.80 (95% CI 1.14 to 20.3) times more likely to have complete tongue base obstructions relative to participants with a lower B3-Fpeak (<1775 Hz; *p* = 0.033).

#### Epiglottitis obstruction

B2-Imax (OR, 1.14, 95% CI 1.02–1.27) was an independent predictor of partial-to-complete epiglottitis obstructions. Participants with a higher B2-Imean (≥66 dB) were 1.14 (95% CI 1.02 to 1.27) times more likely to have partial-to-complete epiglottitis obstructions relative to participants with a lower B2-Imean (<66 dB; *p* = 0.020).

### Patient characteristics, snoring sound parameters, obstruction sites, and surgical response to relocation pharyngoplasty (RP)

Nine (25%) of the participants had a good surgical response. Tonsil obstructions were significantly positively associated with a surgical response to RP, whereas age, AHI, mean SaO_2_, minimal SaO_2_, lateral oropharyngeal wall obstruction, total-, B1-, B2-, and B3-Imeans, and B3-Fpeak were significantly inversely associated with a surgical response (all *p* < 0.05, data not shown).

#### DISE and surgical response

Tonsil obstructions (OR, 21.42, 95% CI 1.62–282.8) and AHI (OR, 0.94, 95% CI 0.89–0.99) were independent predictors of a surgical response (Table 6). Participants with partial-to-complete tonsil obstructions were 21.42 times more likely to have a surgical response relative to participants without DISE-defined tonsil obstructions after adjusting for AHI (*p* = 0.020). Participants with a higher AHI (≥35.4 events/h) were 0.52 (95% CI 0.01 to 0.58) times more likely to have a surgical response relative to participants with a lower AHI (<35.4 events/h) after adjusting for tonsil obstructions (*p* = 0.016).

#### Snoring sound analysis and surgical response

B2-Imean (OR, 0.75, 95% CI 0.59–0.96) and AHI (OR, 0.95, 95% CI 0.91–1.00) were significant predictors of a surgical response after control of B3-Imean and B3-Fpeak. Participants with a higher B2-Imean (≥45 dB) were 0.07 (95% CI 0.01 to 0.52) times more likely to have a surgical response relative to participants with a lower B2-Imean (<45 dB) after adjusting for AHI (*p* = 0.010). Participants with a higher AHI (≥35.4 events/h) were 0.16 (95% CI 0.03 to 0.99) times more likely to have a surgical response relative to participants with a lower AHI (<35.4 events/h) after adjusting for tonsil obstructions (*p* = 0.049). B2-Imean was more specific than AHI for predicting a surgical response.

## Discussion

In this prospective study of OSAHS patients undergoing RP, the velopharynx, oropharynx, and tongue base were the three most frequently encountered sites of obstruction. More than 40% of the participants had multi-level obstructions that were associated with BMI and B1-Imax. Other specific snoring sound parameters such as B2-SI, B2-Imean, B2-Imax, B3-Fmean, and B3-Fpeak were also important markers for velopharynx, tonsil, epiglottis, lateral oropharyngeal wall, and tongue base obstructions, respectively. Patients with a lower AHI and lower B2-Imean or partial-to-complete tonsil obstruction had a better chance of a good surgical response. However, multi-level obstruction as defined by DISE was not a contra-indication for RP since it was not statistically significantly related to a surgical response. In contrast, patients with a lower AHI and lower B2-Imean or partial-to-complete tonsil obstruction had a better chance of a good surgical response. Of note, B2-Imean was also an inverse predictor of tonsil obstruction, meaning that tonsil obstruction defined by snoring sound analysis or DISE rather than by conventional Friedman’s anatomic system was a key factor for a surgical response with RP. These results highlight the importance of a more complex approach to snoring sound analysis accompanied with PSG to determine obstruction sites and select the optimal treatment modality, and suggest that analysis of complex snoring sounds could be combined with PSG to enhance clinical usage.

In this section we discuss three representative examinations evaluating upper airway obstructions during sleep including upper airway pressure measurements, MRI, and DISE. Upper airway pressure measurement with dynamic image recording or airflow monitoring provides a relatively accurate way to record dynamic changes of obstruction sites over a whole night. However, this method is not comfortable and may disturb the patient’s sleep^16^,^19^. Sleep three-dimension MRI has revealed that a severe reduction in retropalatal and retroglossal areas limits airflow and causes mixed-type snoring events^20^, however it is mostly limited to research use due to its expense.

DISE has been widely applied to detect areas of vibration and collapse for surgical planning and surgically failure reason, to adjust pressure with CPAP titration, and to fit a functional mandibular advancement device^21^,^22^,^23^. Even though the validity and reliability of DISE has been confirmed, standards of anaesthetic agents, sedation levels, and scoring methods are still under debate. Some researchers suggest that the range of bispectral index (BIS) within which apnea occurs could be determined for individual patents and applied as a reference for DISE^24^. We recently reported that DISE under BIS-guided propofol infusion, and especially a level of 65–75 [S2 sleep stage, light sleep]^25^, offers an objective and reproducible method to evaluate upper airway collapsibility^26^. However, DISE cannot provide airway information in S3 sleep stage or rapid eye movement stage. Accordingly, DISE findings are not comparable to normal sleep that characterizes by a cyclical pattern of sleep stages (resulting in a change in the tone of muscles of the upper airway) and the change of body position.

Moreover, DISE seems to show additional obstruction which does not need to be modified during upper airway surgery^24^. DISE using the Pringle and Croft classification^18^ cannot be recommended as a reliable predictor of surgical outcome^27^. Moreover, our findings also support a recent systemic review which indicated that DISE-defined epiglottic obstructions occur in 9.7–73.5% of patients with OSAHS, that they can be isolated or combined with other level obstructions, and that they seem to be unrelated to surgical response^28^. Multi-level obstructions are commonly noted during DISE, however this does not affect surgical success.

Since there are some minor limitations of DISE, acoustic analysis of snoring sounds representing a non-contact, non-invasive, inexpensive technology has been proposed to indirectly locate the sites and degree of upper airway obstructions during sleep. Full-night monitoring of snoring sounds may allow to detect the sites of upper airway obstruction that vary continuously, dynamically, with the alternation of stages of natural sleep. For example, B3-Fpeak is an indicator for complete tongue base obstruction. This is comparable with the finding that the mean peak frequencies from 800 Hz to 2000 Hz of the first snoring sounds after lower level obstructive apnea are higher than those after upper level obstructive apnea^19^. Using psychoacoustic algorithms, velar snoring has been shown to be rougher (rapid amplitude modulation, 15–300 Hz) than tonsillar snoring during DISE^29^, and also that post-apnoeic snoring has the largest fluctuation (slower amplitude modulation, <20 Hz). In this study, we confirmed that multi-level obstructions caused apnea during DISE, however we found that they can be predicted by B1-Imax in natural sleep without detecting the strength of fluctuation. Although tonsillar snoring has the highest sharpness (high-frequency signal)^29^, tonsil obstruction is inversely related to B2-Imean. Moreover, we also find that tonsil size was significantly associated with total-SI (*r* = −0.34, *p* = 0.041), B1-SI (*r* = −0.44, *p* = 0.007), and total-Fmean (*r* = 0.36, *p* = 0.031) and statistically insignificantly related to partial-to-complete tonsil obstruction (*r* = 0.25, *p* = 0.15). These findings suggested that tonsil size determined by physical examination and tonsil obstruction defined by DISE were associated with different profile of snoring sound.

It has been reported that some levels of the upper airway primarily obstruct, lower critical pressure, and induce secondary obstruction of other levels^30^. In OSAHS patients with large tonsils and lower tongue position without complete, concentric retropalatal obstruction, pharyngeal surgery such as uvulopalatopharyngoplasty and its modifications (e.g. RP^31^ and extended uvulopalatal flap^32^) can be alternative treatment when nasal CPAP treatment is not tolerable or preferred. However, neither awake modified Mallampati position (MMP) grade nor tongue base dorsalisation is significantly related to DISE-defined tongue base obstruction^33^. For example, nonresponders to pharyngeal surgery often have tongue base obstructions according to postoperative DISE results^22^. In contrast, we found that 67% (6/9) of responders to RP had preoperative tongue base obstructions defined by DISE. We further identified tonsil obstruction defined by either DISE or B2-Imean as an independent predictor of surgical response. According to the concept of fluid dynamics, removing the primary obstruction site can reduce negative pressure and prevent secondary obstruction from occurring^30^. Therefore, 25% (6/24) of tongue base obstructions, 14% (2/14) of epiglottitis obstructions, and 13% (2/15) of multi-level obstructions were secondary, since they were not directly modified by RP. However, further studies are needed to confirm this observation.

The strengths of this study are the complete, multiple evaluations including clinical examination, PSG, full-night snoring sound analysis, DISE, and surgical response. Importantly, diverse snoring sound variables were significantly related to obstruction sites and a surgical response. Furthermore, these variables could predict single obstruction sites, multi-level obstructions, and surgical response with modest-to-high sensitivity but low-to-modest specificity. However, the frequency spectrum of natural snoring sounds during sleep and upper airway obstruction sites identified during DISE may be not comparable with accuracy. Moreover, the small sample size limited the detection of effect sizes for subgroup analysis. Nemes *et al*. found that logistic regression may overestimate odds ratios in studies with small to moderate samples size^34^. In addition, the study lacked a control group. Therefore, the reduction in the AHI may have been the result of other non-surgical factors that could not be controlled. Accordingly, caution should be practiced in the interpretation of the results from the present study.

In summary, our results extend and support findings from previous studies evaluating associations between snoring sounds and obstruction sites. Although complete PSG continues to be useful in research and definitive diagnosis^4^, full-night snoring sound analysis can be: (1) an auxiliary method of PSG to objectively score the severity of snoring; (2) a screening tool to detect different obstructive sites; and (3) a supplementary tool to DISE to determine the sites of secondary obstruction. We recommend that complex snoring sound recording and analysis should be considered to be implemented in acoustic screening devices or added to PSG in the future. At least, intensity and frequency of snoring sounds should be measured in PSG. We could use high maximal intensity of low-frequency snoring sounds (≥60 dB) as a specific surrogate of multi-level obstructions, and low mean intensity of mid-frequency snoring sounds (<45 dB) as a good predictor of a surgical response. Continued analysis of snoring sounds in prospective studies will further aid clinical utility.

## Methods

### Ethics statement

We conducted a prospective case series focusing on patients with OSAHS. Ethics approval was granted by the Institutional Review Board of the Linkou-Chang Gung Memorial Hospital (CGMH), Taoyuan, Taiwan (No. 98–1847A3), which followed the tenets of the Helsinki Declaration. All procedures were in compliance with the current regulations. All participants provided written informed consent.

### Study population

Patients with habitual snoring and witnessed sleep apnea who sought surgical treatment were evaluated prospectively at the Sleep Center, Linkou-CGMH, a tertiary referral centre in Taiwan, between January 2010 and December 2011. The inclusion criteria were: (1) age 20 to 60 years; (2) oropharyngeal narrowing; and (3) willing to participate in this study. Primary exclusion criteria were: (1) gross maxillary and mandibular deformities; (2) history of upper airway surgery for OSAHS or oropharyngeal tumours; and (3) history of allergy to propofol, haemorrhagic disorder, cardiovascular disease, stroke, or morbid obesity (BMI > 35 kg/m^2^). An additional exclusion criterion was an AHI ≤ 5 events/hour. During the study period, 36 participants underwent RP and completed follow-up PSG, 32 of whom have had their baseline variables and acoustic factors described elsewhere^35^. Subjective snoring severity was assessed using a visual analogue scale from 0 (no snoring) to 10 (very severe snoring)^5^, and daytime sleepiness was assessed used the Epworth Sleepiness Scale^36^. Data on age, gender, BMI, neck circumference, tonsil size, MMP, and Friedman’s anatomic stage^14^ were collected.

### Sleep study

All participants underwent attended full-night PSG (Nicolet UltraSom System, Madison, WI, USA) in the sleep laboratory to document sleep parameters. Apnea (defined as a drop in the peak thermal sensor excursion by at least 90% of baseline for at least 10 seconds) and hypopnea (defined as a decrease ≥ 30% in the nasal pressure signal excursions for at least 10 seconds accompanied by desaturation of 4% or more from pre-event baseline or an arousal from sleep) were recorded^37^. AHI, mean SaO_2_ and minimal SaO_2_ were recorded for further analysis. Baseline and follow-up PSGs were performed within 1 month before surgery and 1 year after surgery.

### Automated acoustic analysis of full-night snoring sounds

A non-contact microphone positioned 100 cm above the patient’s head was used to record snoring sounds during PSG examinations as described previously^5^,^35^,^38^,^39^. Six-hour snoring sounds were recorded at a sample rate of 44100 Hz. The frequency power spectrum from 40 Hz to 2000 Hz was formed using fast Fourier transformation. For a full-night analysis of snoring signals, an automatic detection algorithm is implanted base on two criteria: (1) sound energy higher than 0.05 au and (2) sound duration between 0.6 second and 4.0 second^5^. There were four frequency bands (total, 40–200 Hz; B1, 40–300 Hz; B2, 301–850 Hz; and B3, 851–2000 Hz). We analysed each snore and obtained the following variables: (1) SI (events/hour); (2) Imax (dB); (3) Imean (dB); (4) Fpeak (Hz); and (5) Fmean (Hz). Each acoustic parameter was averaged for all detected episodes. Snoring sound signals were analysed using specially designed software (Snore Map^®^, Chang Gung Memorial Hospital, Taoyuan, Taiwan).

### DISE

DISE was performed in a bronchoscopy unit equipped with standard anaesthetic monitoring (oxygen saturation, non-invasive blood pressure, and electrocardiography). The depth of sedation was monitored using an A-2000 BIS-Vista monitor (Version 3.11, Aspect Medical Systems, Inc., Newton, MA). The patients were injected with propofol (10 mg/mL, AstraZeneca, Caponago, Milano, Italy) in the supine position by a pulmonologist with an initial dose of 0.5 mg/kg via a syringe pump (Injectomat Agilia, Fresenius Kabi, France). Another 10–20 mg was given every 30 seconds to meet the target level of sedation (BIS value: 65–75)^25^. The narrowest end inspiratory condition among five consecutive breaths was recorded under BIS-guided propofol infusion^26^. The same authors (Y.L.L. & Y.L.N.) who were blinded to PSG and acoustic analysis performed all DISE examinations and documented the findings.

Four levels of the upper airway were assessed: velopharynx, oropharynx, tongue base, and epiglottis (VOTE system)^40^. The degree of obstruction of the velopharynx or oropharynx was defined as: patent (0–70% obstruction), partial (71–99% obstruction), and complete (100% obstruction). Obstruction of the tongue base was defined as patent (completely or partially visible vallecular), partial (touching the epiglottitis), and complete (pushing the epiglottitis backward). Obstruction of the epiglottis was defined as patent (completely or partially visible vocal cords), partial (no visible vocal folds), and complete (touching the posterior pharyngeal wall). Definitions of obstruction site and degree have been described elsewhere^26^.

Because patients with severe multi-level obstructions seldom respond to a single treatment except for CPAP therapy, we arbitrarily defined ‘at least two sites of complete obstruction’ as ‘multi-level obstructions’, and ‘primary velopharynx (either partial or complete) obstruction with or without another site of partial obstruction’ as ‘simple velopharynx obstruction’.

### RP

This surgery is designed to excise the tonsils, remove supratonsillar adipose tissue, splint the lateral oropharyngeal wall, anteriorly advance the soft palate, and excise the redundant, non-muscular part of the uvula^31^. We performed this procedure under general anaesthesia. The patients usually received oxygen supplementation with oximeter monitoring, intravenous fluids, and medications including antibiotics, steroids, and analgesics for a 4-day admission. We defined a surgical response to RP as a ≥50% reduction in the AHI and an AHI reduced to <10 events/hour^23^.

### Statistical analysis

The sample size for this study was estimated using total-Fpeak to discriminate multi-level obstructions and simple velopharynx obstructions as previously reported^19^. Using a two-tailed Mann-Whitney test to calculate the sample size (normal parent distribution; effect size, 1.128; type I error, 0.05; power, 80%), the minimal total sample size was 30. Due to the small sample size of the subgroup in this study, the descriptive statistics of the variables were presented as median and interquartile range. All data were compared using the Mann-Whitney test or Fisher’s exact test as appropriate. The degrees of correlation between snoring sound parameters and obstruction sites and surgical responses were assessed using the Spearman correlation test. Only the variables with significant values (*p* < 0.05) in the Spearman correlation tests were included in logistic regression analysis to ensure the odds ratios and 95% CI of prediction for multi-level obstructions, other level obstructions, and surgical responses. ROC curves were used to determine the optimal cut-off value, sensitivity, and specificity of detecting the obstruction sites. All *p* values were two-sided, and statistical significance was accepted at *p* < 0.05. All statistical analyses were performed using G*Power (version 3.1.5; University Kiel, Germany) and IBM SPSS software (version 23; International Business Machines Corp., Armonk, NY, USA).

## Additional Information

**How to cite this article**: Lee, L.-A. *et al*. Snoring Sounds Predict Obstruction Sites and Surgical Response in Patients with Obstructive Sleep Apnea Hypopnea Syndrome. *Sci. Rep*. **6**, 30629; doi: 10.1038/srep30629 (2016).

## Figures and Tables

**Figure 1:**
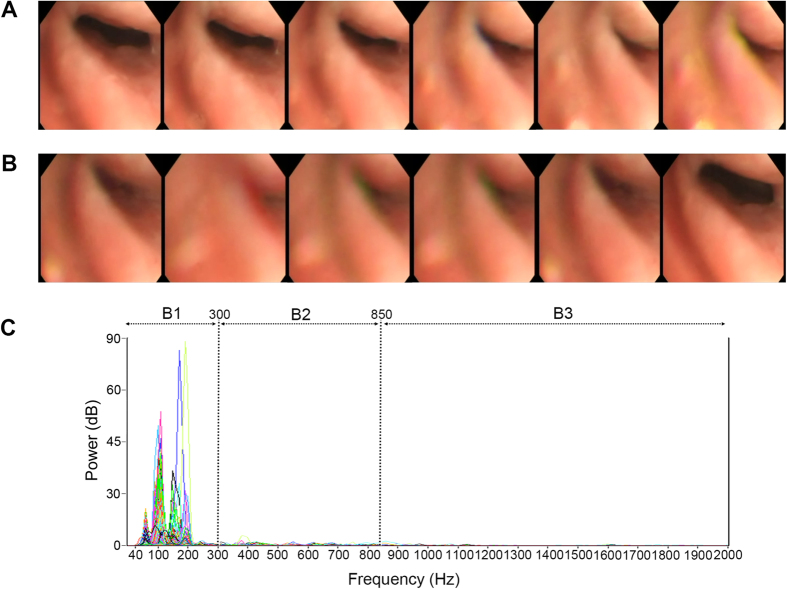
Simple velopharynx obstruction. Demonstration of drug-induced sleep endoscopy and power spectra of full-night snores of a 40-year-old man who had mild obstructive sleep apnea syndrome (apnea-hypopnea index = 14.9 events/hour) and a simple velopharynx obstruction. (**A**) Continuous photographs of the velopharynx revealed that the retropalatal space was narrowing horizontally with anterior-posterior fluttering. (**B**) After complete obstruction, the retropalatal space reopened. (**C**) The power spectra of full-night snores showed that the snoring sounds were loud and centralized in the low-frequency band (B1: 40–300 Hz).

**Figure 2:**
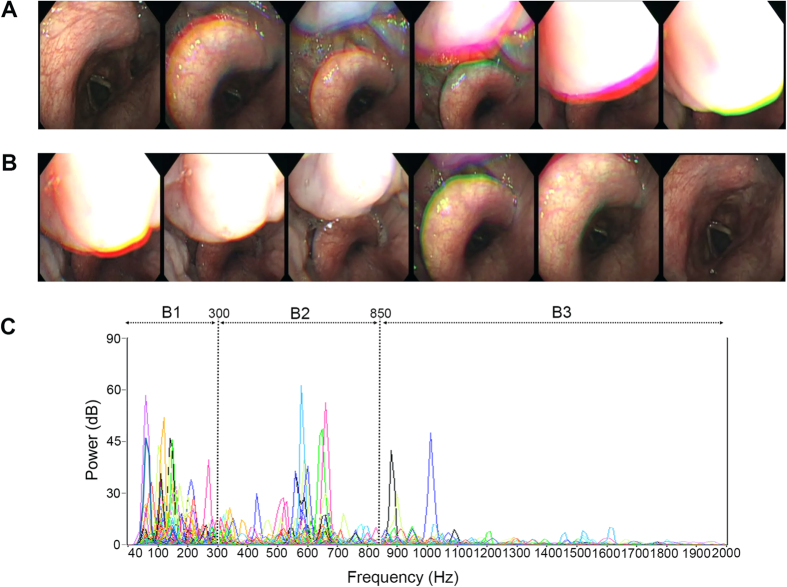
Multi-level obstructions. Demonstration of drug-induced sleep endoscopy and power spectra of full-night snores of a 46-year-old man who had severe obstructive sleep apnea syndrome (apnea-hypopnea index = 62.5 events/hour) and multi-level obstructions (velopharynx, oropharynx, tongue base, and epiglottis). (**A**) Continuous photographs of the epiglottis showed that the epiglottis moved posteriorly and the tongue base gradually dropped. (**B**) After the tongue base had pushed the epiglottitis backward, the completely obstructed airway became patent again. (**C**) The power spectra of full-night snores showed diverse peak sound frequencies in low- (B1: 40–300 Hz), mid- (B2: 301–850 Hz), and high- (B3: 851–2000 Hz) frequency bands with various sound intensities.

**Table 1:** Descriptive characteristics by level of obstruction

	Overall	Level of obstruction		*p* Value^A^
		Multi-level obstruction	Simple velopharynx obstruction	
N (%)	36 (100%)	15 (42%)	21 (58%)	
Age (years)	39 (31, 46)	43 (38, 47)	39 (30, 44)	0.141
Male gender, n (%)	34 (94)	13 (87)	21 (100)	0.167
BMI (kg/m^2^)	26.3 (24.3, 29.0)	28.3 (25.7, 29.8)	25.4 (24.3, 28.2)	0.030
Neck circumference (cm)	39.5 (37.5, 41.0)	40.0 (37.5, 42.0)	39.0 (37.3, 40.0)	0.324
Tonsil size (grade)	2 (2, 3)	2 (2, 3)	2 (2, 3)	0.727
MMP (grade)	2 (2, 3)	3(2, 3)	2 (2, 3)	0.214
FAS (stage)	2 (2, 2)	2 (2, 2)	2 (2, 2)	0.547
Snoring severity (VAS)	8 (6, 9)	8 (6, 9)	8 (6, 9)	0.776
ESS (score)	13 (9, 15)	12 (11, 16)	13 (8, 15)	0.446
AHI (events/hour)	55.9 (27.8, 73.1)	59.88 (53.1, 81.9)	41.3 (25.5, 60.7)	0.021
Mean SaO_2_ (%)	94.0 (91.0, 95.0)	91.0 (85.0, 94.0)	95.0 (93.0, 96.0)	<0.001
Minimal SaO_2_ (%)	76.5 (69.3, 84.8)	69.0 (65.0, 77.0)	80.0 (75.0, 87.0)	0.004

Note: Numbers are median and interquartile ranges, unless otherwise noted as n (%).

^A^*P* Values are based on Fisher exact tests for categorical variables and Mann-Whitney tests for continuous or skewed variables.

AHI, apnea-hypopnoea index; A-stage, anatomic stage; BMI, body mass index; ESS, Epworth Sleepiness Scale; FAS, Friedman’s anatomic stage; MMP, modified Mallampati position; SaO_2_, arterial oxygen saturation; VAS, visual analogue scale.

**Table 2:** Acoustic parameters by level of obstruction.

	Overall	Level of obstruction		*p* Value^A^
		Multi-level obstruction	Simple velopharynx obstruction	
N (%)	36 (100%)	15 (42%)	21 (58%)	
Peak sound frequency (Hz)				
Total (40 Hz–2000 Hz)	1230 (385, 1495)	1340 (1130, 1600)	1130 (255, 1355)	0.025
B1 (40 Hz–300 Hz)	260 (240, 290)	280 (250, 300)	250 (220, 268)	0.008
B2 (301 Hz–850 Hz)	810 (780, 850)	830 (790, 850)	820 (718, 850)	0.170
B3 (851 Hz–2000 Hz)	1910 (1690, 1980)	1940 (1780, 1990)	1785 (1433, 1940)	0.046
Snoring index (events/h)				
Total (40 Hz–2000 Hz)	106 (38, 185)	142 (43, 241)	80 (34, 144)	0.133
B1 (40 Hz–300 Hz)	79 (27, 145)	94 (27, 208)	71 (24, 130)	0.446
B2 (301 Hz–850 Hz)	42 (13, 113)	55 (30, 199)	30 (4, 70)	0.016
B3 (851 Hz–2000 Hz)	51 (5, 143)	72 (25, 174)	34 (1. 81)	0.030
Maximal sound intensity (dB)				
Total (40 Hz–2000 Hz)	71 (65, 77)	74 (70, 78)	67 (62, 76)	0.023
B1 (40 Hz–300 Hz)	61 (56, 65)	63 (61, 66)	58 (51, 62)	0.001
B2 (301 Hz–850 Hz)	65 (59, 69)	69 (65, 72)	60 (56, 66)	0.001
B3 (851 Hz–2000 Hz)	70 (63, 75)	72 (69, 75)	66 (54, 75)	0.053
Mean sound intensity (dB)				
Total (40 Hz–2000 Hz)	52 (46, 57)	54 (50, 58)	48 (45, 53)	0.021
B1 (40 Hz–300 Hz)	45 (40, 49)	48 (42, 51)	40 (38, 48)	0.021
B2 (301 Hz–850 Hz)	49 (42, 52)	52 (46, 53)	48 (40, 51)	0.036
B3 (851 Hz–2000 Hz)	54 (44, 57)	56 (51, 58)	48 (41, 56)	0.131
Mean sound frequency (dB)				
Total (40 Hz–2000 Hz)	177 (128, 310)	254 (143, 361)	167 (120, 272)	0.102
B1 (40 Hz–300 Hz)	122 (104, 146)	122 (110, 147)	119 (101, 146)	0.324
B2 (301 Hz–850 Hz)	452 (418, 517)	439 (384, 522)	459 (420, 511)	0.446
B3 (851 Hz–2000 Hz)	1206 (1094, 1305)	1222 (1180, 1376)	1149 (1060, 1277)	0.133

NOTE: Numbers are median and interquartile range, unless otherwise noted as n (%).

^A^*P*-values are based on Fisher’s exa3ct tests for categorical variables and Mann-Whitney tests for continuous or skewed variables.

**Table 3:** The sites and degrees of obstruction determined by drug-Induced sleep endoscopy.

Classification of obstruction	Overall (*n* = 36)	Degree of obstruction		
		Patency	Partial obstruction	Complete obstruction
Site of obstruction- the VOTE classification^39^				
Velopharynx	36 (100)	0 (0)	18 (50)	18 (50)
Oropharynx	32 (89)	4 (11)	27 (75)	5 (14)
Tonsils	18 (50)	18 (64)	15 (42)	3 (8)
Lateral walls	13 (36)	23 (63)	11 (31)	2 (6)
Tongue base	24 (67)	12 (33)	3 (8)	21 (58)
Epiglottis	14 (39)	22 (61)	14 (39)	0 (0)
Level of obstruction				
Simple velopharynx obstruction	21 (58)	0 (0)	12 (33)	9 (25)
Multi-level obstruction^A^	15 (42)	0 (0)	0 (0)	15 (42)

NOTE: Numbers are n (%).

^A^Patients with at least two sites of complete obstruction.

VOTE, velopharynx, oropharynx, tongue base, and epiglottis.

**Table 4:** Spearman Correlation of Patient Characteristics with Drug-Induced Sleep Endoscopic Findings.

Site	Multi-Level	Velopharynx	Oropharynx		Tongue Base	Epiglottis
	≥2 sits		Tonsils	Lateral Wall		
Degree of obstruction	Complete	Complete	Partial-to-Complete	Partial-to-Complete	Complete	Partial-to-Complete
N (%)	15 (42)	18 (50)	18 (50)	13 (37)	21 (58)	14 (39)
Age	0.25	0.39^A^	−0.32	0.37^A^	0.25	0.20
Male gender,	−0.29	0.00	0.24	−0.32	−0.21	−0.30
BMI	0.37^A^	−0.08	−0.12	0.22	−0.05	0.29
Neck circumference	0.17	0.01	−0.17	0.13	−0.18	0.13
Tonsil size	0.07	0.25	0.25	0.02	−0.10	0.03
MMP	0.23	0.25	0.06	0.05	0.07	0.28
FAS	0.14	−0.06	0.05	−0.11	0.08	0.13
Snoring severity	0.05	0.10	0.04	0.07	0.04	0.04
ESS	0.13	0.16	−0.15	0.33^A^	0.20	0.09
AHI	0.39^A^	0.36^A^	−0.14	0.36^A^	0.15	0.41^A^
Mean SaO_2_	−0.60^A^	−0.38^A^	0.09	−0.33^A^	−0.24	−0.52^A^
Minimal SaO_2_	−0.48^A^	−0.51^A^	0.05	−0.35^A^	−0.29	−0.42^A^

NOTE: Data are the unadjusted Spearman correlation coefficients/adjusted partial correlation coefficients for BMI.

^A^*P*-values are less than 0.05.

AHI, apnea-hypopnea index; A-stage, anatomic stage; BMI, body mass index; ESS, Epworth Sleepiness Scale; FAS, Friedman’s anatomic stage; MMP, modified Mallampati position; SaO_2_, arterial oxygen saturation; VAS, visual analogue scale.

**Table 5:** Spearman correlation of acoustic parameters with drug-induced sleep endoscopic findings

Site	Multi-Level	Velopharynx	Oropharynx		Tongue Base	Epiglottis	
	≥2 Sites		Tonsils	Lateral Wall			
Degree of Obstruction	Complete	Complete	Partial-to-Complete	Partial-to-Complete	Complete	Partial-to-Complete	
N (%)	15 (42)	18 (50)	18 (50)	13 (37)	21 (58)	14 (39)	
Peak sound frequency							
Total	0.37^A^	0.16	−0.13	0.36^A^	0.21	0.31	
B1	0.44^A^	0.33^A^	−0.10	0.28	0.21	0.39^A^	
B2	0.24	0.39^A^	0.13	0.16	0.28	0.16	
B3	0.34^A^	0.29	−0.13	0.30	0.40^A^	0.27	
Snoring index							
Total	0.26	0.09	−0.05	0.11	0.11	0.19	
B1	0.13	−0.01	−0.08	0.08	0.02	0.07	
B2	0.40^A^	0.23	0.06	0.18	0.26	0.33^A^	
B3	0.37^A^	0.35^A^	0.10	0.16	0.33^A^	0.29	
Maximal sound intensity							
Total	0.38^A^	0.00	−0.17	0.24	0.09	0.30	
B1	0.53^A^	0.14	−0.27	0.40^A^	0.18	0.44^A^	
B2	0.55^A^	0.11	−0.23	0.38^A^	0.31	0.46^A^	
B3	0.33	0.05	−0.18	0.27	0.11	0.25	
Mean sound intensity							
Total	0.39^A^	0.11	−0.21	0.38^A^	0.16	0.31	
B1	0.39^A^	0.08	-−0.23	0.30	0.20	0.32	
B2	0.36^A^	0.05	−0.44^A^	0.45^A^	0.21	0.28	
B3	0.29	0.05	−0.22	0.35^A^	0.14	0.24	
Mean sound frequency							
Total	0.28	0.25	0.09	0.15	0.08	0.24	
B1	0.17	−0.03	0.11	0.06	−0.07	0.19	
B2	−0.13	0.10	0.05	−0.17	0.18	−0.10	
B3	0.26	0.09	−0.36^A^	0.40^A^	0.17	0.27	

NOTE: Data are the unadjusted Spearman correlation coefficients.

^A^*P*-values are less than 0.05.

**Table 6:** Predictors for obstruction sites and surgical response.

	Logistic Regression				Receiver Operating Characteristic Curve		
	Predictors^A^	Odds Ratio	95% CI	*p* Value	Cut-Off Value	Sensitivity	Specificity
Obstruction sites							
Multi-level^B^ (*n* = 15)	BMI	1.48	1.02–2.15	0.039	25.6 kg/m^2^	80%	57%
	B1-Imax	1.25	1.05–1.49	0.011	60 dB	93%	67%
Velopharynx^B^ (*n* = 18)	B2-SI	1.01	1.00–1.02	0.045	28 events/h	83%	61%
Oropharynx-tonsils^C^ (*n* = 18)	B2-Imean	0.84	0.74–0.96	0.013	48 dB	78%	67%
Oropharynx-lateral wall^C^ (*n* = 13)	B2-Imean	1.22	1.03–1.46	0.022	48 dB	92%	65%
	B3-Fmean	1.01	1.00–1.02	0.040	1220 Hz	69%	70%
Tongue base^B^ (*n* = 21)	B3-Fpeak	1.00	1.00–1.01	0.016	1775 Hz	76%	60%
Epiglottis^C^ (*n* = 15)	B2-Imax	1.14	1.02–1.27	0.020	66 dB	79%	73%
Surgical response (*n* = 9)							
Defined by drug-induced sleep endoscopy	Tonsil obstruction^C^	21.42	1.62–282.8	0.020	Yes	89%	63%
	AHI	0.94	0.89–0.99	0.025	35.4 events/h	82%	67%
Defined by snoring sound analysis	B2-Imean	0.75	0.59–0.96	0.020	45 dB	82%	78%
	AHI	0.95	0.91–1.00	0.049	35.4 events/h	82%	67%

NOTE: Numbers are median and interquartile range, unless otherwise noted as n (%). CI, confidence interval; Fmean, mean sound frequency; Fpeak, peak sound frequency; Imax, maximal sound intensity; Imean, mean sound intensity; SI, snoring index.

^A^All predictors are continuous variables except for tonsil obstruction (binominal).

^B^Complete obstruction.

^C^Partial-to-complete obstruction.
